# DNA Methylation and HPV-Associated Head and Neck Cancer

**DOI:** 10.3390/microorganisms9040801

**Published:** 2021-04-10

**Authors:** Takuya Nakagawa, Tomoya Kurokawa, Masato Mima, Sakiko Imamoto, Harue Mizokami, Satoru Kondo, Yoshitaka Okamoto, Kiyoshi Misawa, Toyoyuki Hanazawa, Atsushi Kaneda

**Affiliations:** 1 Department of Otorhinolaryngology, Head and Neck Surgery, Graduate School of Medicine, Chiba University, Chiba 260-8670, Japan; tnakagawa121@gmail.com (T.N.); t-kurokawa@chiba-u.jp (T.K.); sakikofujii@gmail.com (S.I.); yokamoto@faculty.chiba-u.jp (Y.O.); 2 Department of Molecular Oncology, Graduate School of Medicine, Chiba University, Chiba 260-8670, Japan; mima-masato@chiba-u.jp (M.M.); h.mizokami@chiba-u.jp (H.M.); ksatoru@med.kanazawa-u.ac.jp (S.K.); 3 Moores Cancer Center, University of California San Diego, La Jolla, CA 92037, USA; 4 Clinical Research Center, Chiba University Hospital, Chiba 260-8677, Japan; 5 Department of Otorhinolaryngology, Head and Neck Surgery, School of Medicine, Hamamatsu University, Hamamatsu 431-3192, Japan; kiyoshim@hama-med.ac.jp; 6 Department of Otorhinolaryngology, Head and Neck Surgery, Graduate School of Medical Science, Kanazawa University, Kanazawa 920-8640, Japan; 7 Chiba Rosai Hospital, Ichihara 290-0003, Japan

**Keywords:** human papillomavirus, head and neck squamous cell carcinoma, oropharyngeal squamous cell carcinoma, DNA methylation

## Abstract

Head and neck squamous cell carcinoma (HNSCC), especially oropharyngeal squamous cell carcinoma (OPSCC), has recently been found to be significantly associated with human papillomavirus (HPV) infection. The incidence of OPSCC has been increasing and surpassed the number of cervical cancer cases in the United States. Although HPV-associated OPSCC has a relatively better prognosis than HPV-negative cancer, approximately 20% of HPV-associated HNSCC patients show a poor prognosis or therapeutic response, and the molecular mechanism behind this outcome in the intermediate-risk group is yet to be elucidated. These biological differences between HPV-associated HNSCC and HPV-negative HNSCC are partly explained by the differences in mutation patterns. However, recent reports have revealed that epigenetic dysregulation, such as dysregulated DNA methylation, is a strikingly common pathological feature of human malignancy. Notably, viral infections can induce aberrant DNA methylation, leading to carcinogenesis, and HPV-associated HNSCC cases tend to harbor a higher amount of aberrantly methylated DNA than HPV-negative HNSCC cases. Furthermore, recent comprehensive genome-wide DNA-methylation analyses with large cohorts have revealed that a sub-group of HPV-associated HNSCC correlates with increased DNA methylation. Accordingly, in this review, we provide an overview of the relationship between DNA methylation and HPV-associated HNSCC.

## 1. Introduction

Head and neck cancer was the 7th most common cancer worldwide in 2018, with approximately 890,000 new cases and 450,000 deaths [1,2]. More than 90% of head and neck tumors are head and neck squamous cell carcinoma (HNSCC), arising from the mucosal surfaces of the head and neck region [3,4]. Remarkably, although the general causes of HNSCC are smoking and alcohol consumption, human-papillomavirus (HPV) infections have also recently been identified to be significantly associated with HNSCC, and especially with oropharyngeal squamous cell carcinoma (OPSCC). This virus is known to be a common cause of cervical cancer. Although the incidence of cervical cancer has been stagnant as a result of the vaccination efforts and novel screening systems [5], the incidence of OPSCC has been increasing and surpassed the number of cervical cancer cases in the United States [6,7]. Furthermore, unlike cervical cancer, there are no screening methods for HPV-associated OPSCC [8], and the incidence is expected to increase until 2060 [9].

HPV-associated OPSCC has different biological and clinical features from HPV-negative HNSCC [10,11]. A meta-analysis has already shown that patients with HPV-associated OPSCC have an approximately 28% lower risk of death than HPV-negative patients [12]. Given these data, the eighth edition of the American Joint Committee on Cancer (AJCC) Staging Manual, Head and Neck Section, updated the separate staging algorithm for high-risk HPV-associated cancer of the oropharynx, because of the distinct features of this cancer, including treatment response and prognosis [13]. To improve the prognosis and overall survival in HPV-associated OPSCC, several clinical trials have tested the effect of treatment de-escalation, aiming to decrease the treatment-induced acute and late toxicity [14]. However, approximately 20% of HPV-associated OPSCC patients have poor prognoses and/or therapeutic responses [15], and the molecular mechanism behind the outcomes in this intermediate-risk group is yet to be elucidated.

These biological differences between HPV-associated HNSCC and HPV-negative HNSCC are partly explained by the differences in mutation patterns. Frequent mutations in several genes, such as *TP53*, *CDKN2A*, and *PIK3CA*, as well as in members of the *NOTCH* pathway, have been reported in HPV-negative HNSCC via genomic and transcriptomic approaches [16,17,18,19]. In HPV-associated OPSCC, p53 and RB are mainly inactivated by HPV E6 and E7 oncoproteins, respectively [19,20,21,22]; therefore, somatic mutations in *TP53* and *CDKN2A* are very rare. However, somatic mutations in *PIK3CA*, *E2F1*, and *TRAF3* have been reported [23], and chromatin regulators, such as lysine methyltransferase2C (*KMT2C*), *KMT2D*, CREB-binding protein (*CREBBP*), and E1A-associated protein p300 (*EP300*), are also mutated in HPV-associated OPSCC [24]. In addition, apolipoprotein B mRNA-editing catalytic polypeptide-like (APOBEC)-mediated mutagenesis, such as *PIK3CA* mutation and HPV genome mutation, has been reported in HPV-associated HNSCC [25,26,27]. The APOBEC3 signature is also displayed in HPV-associated HNSCC [25,26,27]. Overall, HPV-associated OPSCC shows relatively fewer genetic alterations in cancer drivers than HPV-negative tumors at the exome level [18].

Apart from mutations, epigenetic dysregulation is also a common pathological feature in human malignancy [28,29,30,31]. HNSCC samples have been characterized according to their patterns of DNA methylation, one of the critical epigenetic mechanisms that silence tumor suppressor genes in cancers [25]. In particular, it has been reported that viral infections can induce aberrant DNA methylation during carcinogenesis [32,33], and HPV-associated HNSCC tends to harbor a higher amount of aberrantly methylated DNA than HPV-negative HNSCC [34,35]. This review provides an overview of the relationship between DNA methylation and HPV-associated HNSCC.

## 2. DNA Methylation and Its Association with Cancer

Epigenetics was first defined in 1942 by Conrad Waddington [36] and is a mechanism that regulates gene expression without changing the DNA sequence. DNA methylation is a major epigenetic mechanism that comprises the direct modification of DNA via the addition of a methyl group to the 5′-positions of the cytosines within CpG dinucleotides to form 5-methylcytosine (5mC). DNA methylation is a well-recognized means to regulate gene expression. CpG islands, which are regions of >500 bp and with a GC content >55%, are mainly located in the promoter regions and kept free of methylation [37]. However, aberrant de novo methylation of CpG islands is a hallmark of human cancers and is found early during carcinogenesis [38].

**Figure 1:**
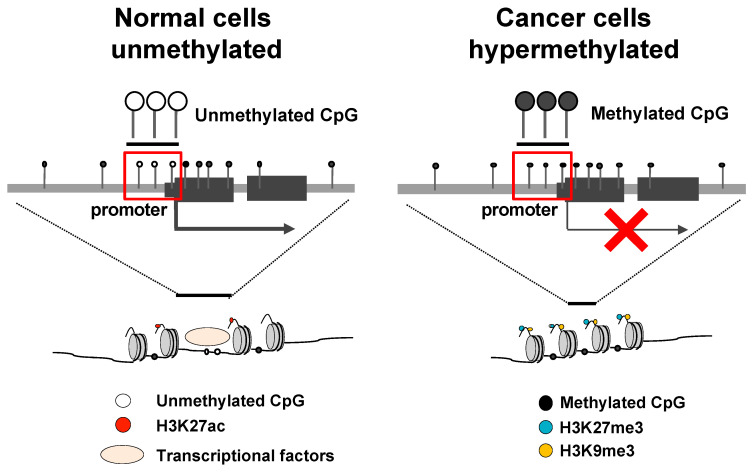
Transcriptional repression by promoter DNA hypermethylation and histone modifications. In normal cells, the CpG islands of promoter regions are open chromatin and generally unmethylated. Gene transcription is activated by transcriptional factors with H3K27ac (**left**). In cancer cells, the CpG islands in the promoter regions are hypermethylated, constructing heterochromatin with H3K27me3 and H3K9me3, which suppresses gene transcription (**right**). Abbreviations: H3K27me3, trimethylation of lysine 27 on histone 3; H3K9me3, trimethylation of lysine 9 on histone 3; H3K27ac, acetylation of lysine 27 on histone 3.

DNA methyltransferases (DNMTs) can induce DNA methylation [39]. They transfer a methyl group from S-adenyl methionine (SAM) to the fifth carbon of a cytosine residue to form 5mC [39]. Transcriptionally repressed (heterochromatic) regions, which are enzymatically inaccessible cis-regulatory elements, are characterized by methylated DNA and specific histone modifications, such as trimethylation of lysine 27 on histone 3 (H3K27me3) [40,41] and trimethylation of lysine 9 on histone 3 (H3K9me3) [35,36] (Figure 1). Aberrant DNA methylation can be repaired via meiosis but is heritable [42,43]. DNA methylation is widely known to affect carcinogenesis [38,44]. Aberrant DNA hypermethylation of promoter regions is a major mechanism for silencing tumor suppressor genes in various cancers [45]. In addition, these DNA methylations could be used as prognostic biomarkers in cancer due to their potential to predict prognosis and/or response to therapy [46].

## 3. Virus-Associated Carcinogenesis and DNA Methylation

Aberrant DNA methylation in cancers is associated with prior viral infections. As a result of host defense against the virus invasion, DNA methylation of the viral genome might be induced [47]. However, viral infections also induce aberrant DNA methylation in the human genome, leading to carcinogenesis [32,33]. The relationship between virus-associated cancers and DNA methylation, such as that in gastric cancer associated with Epstein–Barr virus (EBV) [48], hepatocellular carcinoma associated with the hepatitis B virus (HBV) and hepatitis C virus (HCV) [49], and cervical cancer [50] and HNSCC, both associated with HPV [23], have been reported, and these aberrant DNA methylation events could be induced by viral infections. For example, in gastric cancer, approximately 10% of the gastric cancer cases are EBV (+) gastric cancers, showing higher levels of DNA methylation compared to those in EBV (−) gastric cancers [48,51]. Latent membrane protein 2A (LMP2A), which is one of the EBV proteins, activates STAT3, and upregulates *DNMT1* [52,53]. In hepatocellular carcinoma, HBV protein X (HBx) upregulates *DNMT* genes directly and recruits DNMTs to target genes, such as *IL-4R* and *MT1F* [53,54,55].

## 4. HPV and DNA Methylation

HPV is a small, circular, double-stranded virus that targets the basal layer of the epithelial cells [14,56]. In the head and neck region, HPV targets the oropharynx, especially the tonsils, and the base of the tongue. There are more than 200 HPV types, which can be divided into high-risk and low-risk types based on their potential to induce cancer [57]. Persistent high-risk HPV infections can progress to invasive cancer within 10 years, although the majority of these infections are cleared within 1 or 2 years [58,59]. In HNSCC, >90% of HPV-associated cases involve HPV16, which is classified as a high-risk HPV [60].

HPV16 is approximately 7900 bp in size. It exists in the nucleus of infected cells as a circular episome. The HPV16 genome contains several genes that encode proteins that are transcribed following the differentiation of the infected cells [61,62]. The proteins produced early during the infection are known as early proteins: E1, E2, E4, E5, E6, and E7. The proteins produced late during the infection are known as late proteins: L1 and L2 [63,64]. There is a long control region that codes no protein between the L1 stop codon and E6 AUG, and it contains the early viral promoter p97. Another promoter p670 that is related to the late viral promoter exists in the E7 coding region [62]. Based on these two promoters, HPV16 oncoproteins are generated. Among these HPV proteins, E2 inhibits the p97 promoter and results in inhibition of E6 and E7 [62,65]. Therefore, inhibition of E2, such as E2 disruption caused by HPV genome integration to human genome or DNA methylation of E2 binding site, causes the upregulation of E6 and E7 [66]. As mentioned herein, although HPV E6 and E7 are oncoproteins and inactivate p53 and RB respectively, these proteins also regulate the DNA methylation of the host genome.

**Figure 2:**
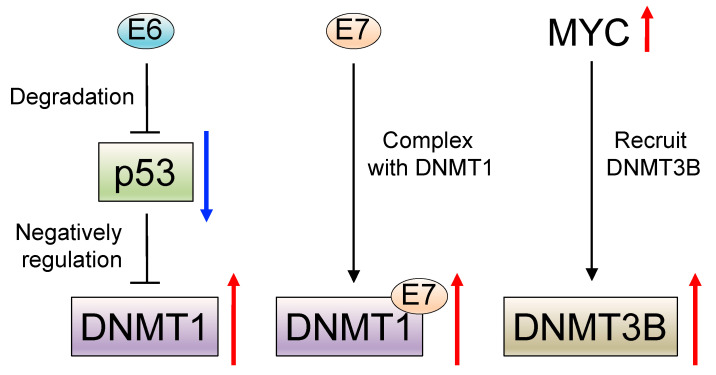
The molecular mechanism of induction of DNA methylation in human papillomavirus (HPV)--associated head and neck squamous cell carcinoma (HNSCC). Wild-type p53 negatively regulates DNA methyltransferase 1 (DNMT1). HPV E6 oncoprotein cause degradation of p53 and it results in the upregulation of DNMT1 (**left**). HPV E7 oncoprotein forms a complex with DNMT1 and results in DNMT1 upregulation (**middle**). MYC is upregulated in HPV-associated HNSCC and recruit DNMT3B (**right**).

For example, wild-type p53 negatively regulates DNA methyltransferase 1 (DNMT1) expression by forming a complex with specificity protein 1 (Sp.1) and chromatin modifiers on the DNMT1 promoter in lung cancer [67]. In HPV-associated HNSCC, degradation of p53 is generally caused by the HPV E6 oncoprotein [19,20,21,22], and DNMT1 is consequently upregulated [68]. Additionally, the HPV E7 oncoprotein has been reported to form a complex with DNMT1 [69,70] and DNMT1 is upregulated in HPV-associated OPSCC [71] and cervical cancer [72]. Apart from E6 and E7, *c-Myc* (MYC) is also reported to recruit DNA methyltransferase 3 alpha (DNMT3A) [73], and the MYC-associated genetic network is reported to be activated in HPV-associated HNSCC [74,75,76] (Figure 2).

## 5. DNA Methylation and HPV-Associated HNSCC

Many studies focusing on DNA methylation of various genes in HPV-associated HNSCC have been reported. The differentially methylated genes in HPV-associated HNSCC are involved in cell-cycle regulation (*CCNA1* [34,77,78]), apoptosis (*RASSF1* [79]), cellular adhesion (cadherin genes, such as *CDH1*, *CDH8*, *CDH11*, *CDH13*, *CDH15*, *CDH18*, *CDH19*, and *CDH23* [78,80,81,82,83], and *ITGA4* [80,84]), cellular migration (*TIMP3* [82]), differentiation (*CTNNA2*, *RXRG,* and *GATA4* [80,85]), and G protein-coupled receptor (GPCR) genes (*GHSR*, *CASR*, *NMUR1*, *PTGDR1*, *PTGDR2*, and *PTGIR* [80,86,87]) (Table 1). These analyses revealed that DNA methylations represented useful prognostic or treatment response markers. 

DNA methylation analysis of a small-sample cohort on a genome-wide scale has also been reported (Table 2). Lechner et al. performed a genome-wide DNA methylation analysis of 32 formalin-fixed paraffin-embedded OPSCC samples [18 HPV (+) and 14 HPV (–)] using the Infinium 450 k bead array and showed that polycomb repressive complex 2 target genes, including *CDH1*, are hypermethylated in the HPV (+) cases [81]. Parfenov et al. studied the DNA methylation profiles of 35 HPV (+) HNSCC cohorts from TCGA (including 22 OPSCC cases) and reported that *BARX2* and *IRX4* are hypermethylated and repressed in integration-negative tumors [88]. Nakagawa et al. performed Infinium 450 k bead array analysis of 13 OPSCC samples [4HPV (+) and 9 HPV (−)] and four normal mucosal samples and identified that hypermethylation of *RXRG*, *GHSR*, *CTNNA2*, and *ITGA4* are significantly correlated with HPV (+) status [80]. The methylation status of these genes was validated in 70 HNSCC samples by pyrosequencing. Among them, *RXRG* methylation showed a significant correlation with a favorable prognosis. Kostareli et al. performed DNA methylation analysis using a human CpG-island tiling array for five HPV (+) and 10 HPV (−) HNSCC cases and reported that *ALDH1A2, OSR2, GATA4*, *GRIA4,* and *IRX4* were significantly hypermethylated in the HPV (+) HNSCC samples [85]. There were significant correlations between DNA methylation levels and the expression of these five genes, among which low methylation of *ALDH1A2* and *OSR2*, and high methylation of *GATA4*, *GRIA4*, and *IRX4* were correlated with favorable prognosis.

In these reports, although some of the markers were shared among several cohorts, there were many cohort-specific DNA methylation markers. This observation is partly because of the small sample size and the difference in the methods used for the analyses, such as probe position or the selection of differentially methylated regions.

## 6. Comprehensive Large-Cohort DNA Methylation Analysis at a Genome-Wide Scale

Recently, several comprehensive large cohort analyses have been reported (Table 3). The Cancer Genome Atlas (TCGA) consortium performed comprehensive DNA methylation and genomic analyses on a genome-wide scale for 279 HNSCC samples, including 36 HPV (+) HNSCC samples [23]. Genome-wide analysis using Infinium 450 k bead array demonstrated the existence of several DNA methylation subtypes including CpG island methylation. However, its association with HPV status was not significant in this cohort. Esposti et al. combined their Infinium 450 k bead array data on 12 HNSCC samples, including 6 HPV (+) samples, with the TCGA data [23] and data from the University College London (UCL) Cancer Institute [81] mentioned previously herein [a total of 326 HNSCC cases, including 63 HPV (+) cases]. The authors performed unsupervised clustering analysis using 2410 differentially methylated positions and observed distinct DNA methylation patterns in the HPV (+) samples [89]. Papillon-Cavanagh et al. re-analyzed the updated TCGA data on 528 HNSCC samples, including 99 HPV (+) HNSCC samples. They classified HNSCC samples into five groups: one HPV (+) and four HPV (−) groups [90]. In HPV (−) groups, there was one cluster named lysine 36 on histone 3 (H3K36) cluster, with H3K36 Met alteration or *NSD1* mutation. The other three groups were not precisely described. Taken together, these data suggested that at least HPV-associated HNSCC had a distinct DNA methylation pattern compared to HPV-negative HNSCC. However, the HPV (+) cases could not be further stratified because of the analysis of many HPV (−) cases in these reports.

Ren et al. performed MBD-seq on 50 HPV+ OPSCC samples and 25 healthy mucosal tissues and identified 20 highly specific differentially methylated regions in HPV (+) OPSCC compare with normal mucosa [84]. Ando et al. explored the MBD-seq results of 47 HPV (+) HNSCC and 25 healthy mucosal samples, among which selected 59 genes that showed significant negative correlations between DNA methylation and RNA expression. The authors performed unsupervised hierarchical clustering of these genes and stratified the HPV (+) samples, which revealed a high-DNA methylation phenotype in HPV (+) cases [91]. Additional clustering analysis of the same 59 genes but in the Infinium 450 k bead array data of TCGA 54 OPSCC samples showed the same high-DNA methylation phenotype. Additionally, they observed that the histone acetyltransferase *CREBBP* was significantly mutated in the group with increased DNA methylation.

**Figure 3:**
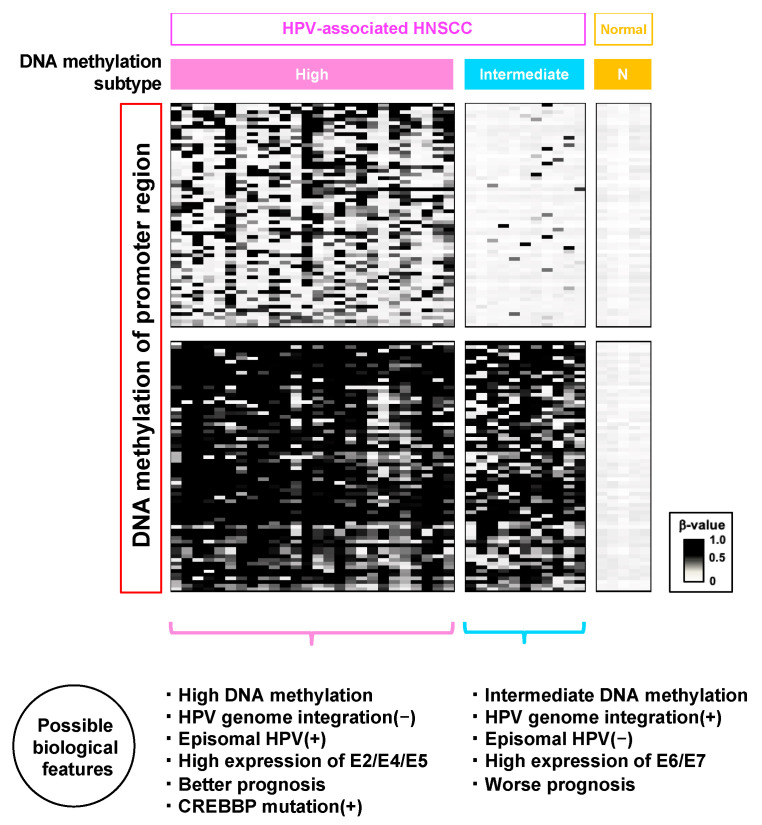
Distribution of DNA methylation pattern and biological characteristics in HPV-associated OPSCC. Genome-wide DNA methylation analysis revealed two types of DNA methylation patterns in HPV-associated OPSCC. In high DNA methylation subtype, HPV genome integration (−), episomal HPV (+), high expression of E2/E4/E5, and better prognosis compared to the intermediate DNA methylation subtype and *CREBBP* mutation (+) were previously reported. In intermediate DNA methylation subtype, HPV genome integration (+), episomal HPV (−), high expression of E6/E7, and worse prognosis compared to the high DNA methylation subtype were reported.

Nakagawa et al. also identified a high-DNA methylation subtype in HPV-associated OPSCC and showed that this subtype was correlated with a good prognosis. They performed Infinium 450 k bead array analysis on 89 OPSCC samples in combination with 81 OPSCC samples from TCGA [83]. Unsupervised hierarchical clustering using 1315 probes targeting the promoter region showed that HPV (+) OPSCC was stratified into two epigenotypes, reflecting different clinicopathological features. The HPV (+)-high methylation phenotype showed the most favorable outcome among the generally favorable HPV (+) OPSCC cases. Although some patients with HPV-associated OPSCC exhibit therapeutic resistances and/or poor prognoses, the authors stratified those groups by epigenetic subtypes that could not be achieved based on other features such as genomic mutations. In this analysis, the authors also performed targeted exon sequencing, but there was no correlation between genetic mutations including *CREBBP* and DNA-methylation subgroups in both of their cohorts and the TCGA dataset. Given these data, an HPV-associated OPSCC subtype with increased DNA methylation has only recently been discovered by two different research groups, although further analysis of the characteristics of this subgroup is needed (Figure 3).

## 7. What Induces High DNA Methylation Subtypes?

It is still difficult to elucidate what induces these different methylation patterns, especially the subtype with increased DNA methylation. By examining a cohort of 35 HPV (+) HNSCC cases from TCGA, Parfenov et al. reported that HPV-integration (−) HNSCC showed relatively higher methylation than HPV-integration (+) HNSCC. In addition, the DNA methylation status of HPV-integration (+) HNSCC is similar to that of HPV-negative HNSCC [88]. This observation suggests that HPV-integration (−) tumors are of the high-DNA methylation subtype. Although there was no significant association found between the HPV integration statuses and clinical outcomes in this cohort due to the shortage of samples, Nulton et al. reported a significant correlation between HPV-integration (+) status and poor survival in 56 HPV-associated HNSCC cases [92].

In HPV-associated cervical cancer, the TCGA performed DNA methylation analysis of 178 primary cervical cancers including 169 HPV (+) cases at a genome-wide scale using the Infinium 450 k bead array [50]. In this analysis, there were three DNA methylation subtypes, including a group with a hypermethylated CpG island (CIMP-high), which is related to the adenocarcinoma cluster. This adenocarcinoma cluster is always associated with the integration (+) status and is mainly caused by HPV18, which is in clade A7. HPV integration into the human genome is related to the loss of E2 expression. Gagliardi et al. also performed DNA methylation analysis on a genome-wide scale on 118 cervical cancer samples using an 850 k EPIC array and also detected a clade-specific DNA methylation pattern [93]. However, only approximately 76% of HPV16, which is a clade-A9-related cancer, was related to the integration (+) status. HPV integration (−) indicates the presence of episomal HPV, and the presence of episomal HPV is associated with active HPV infection and epithelial differentiation [94].

These data are consistent with the fact that HPV16-induced cancers, such as HPV-associated HNSCC, have two different types of HPV infection patterns—HPV integration (+) or HPV integration (−/episomal HPV (+), as described previously herein. In this analysis, although the authors did not study the difference in DNA methylation pattern between the two infection patterns in HPV16-associated cancer, there is a possibility that the integration status changes the DNA methylation pattern in both HPV16-related cervical cancer and HNSCC.

In HPV-associated HNSCC, Ren et al. reported that different expression patterns of HPV genes are correlated with the integration status of the virus [95]. In 69 HPV-associated HNSCC cases in the TCGA dataset, those with HPV integration (+) disease showed a high expression of E6/E7 and low expression of E2/E4/E5, whereas those with HPV integration (−) disease showed a high expression of E2/E4/E5 and low expression of E6/E7. The authors validated this result by using an independent HPV-associated OPSCC cohort and TCGA cervical cancer dataset. When the HPV genome integrates into the human genome, E2 is generally disrupted, followed by the upregulation of E6/E7 [96,97,98]. From this point of view, these results are consistent with those of previous reports. Although further analysis is needed, these differences in the expression patterns of HPV genes might be related to the different DNA methylation patterns observed in HNSCC subtypes.

## 8. Circulating Tumor DNA Methylation

DNA methylation analysis is also available from blood samples, based on around 200 base pairs of circulating tumor DNA (ctDNA) [99]. Liquid biopsy technology using this ctDNA is useful for detecting cancer less invasively. Moreover, ctDNA can be used for assessing diagnostic markers but also for prognostic or monitoring markers. However, detecting mutations using ctDNA requires high sequence depths and is associated with high costs, which can represent an additional challenge in the assessment of some genes, such as *TP53* or *NOTCH1*, which harbor mutations also in multiple exons [100]. In addition, the mutation is known to be a later event during carcinogenesis than DNA methylation; thus, DNA methylation analysis has advantages over mutation determination in liquid biopsies [101,102]. ctDNA analysis using HPV-associated OPSCC samples has been previously performed. Misawa et al. performed quantitative methylation-specific PCR of *CALML5*, *DNAJC5G*, and *LY6D* for ctDNA samples from eight HPV-associated OPSCC [103]. Pre-treatment ctDNA samples showed high methylation levels in these three genes (100%, 87.5%, and 87.5%), whereas post-treatment ctDNA samples showed lower methylation levels (25%, 0%, and 12.5%, respectively) [103], suggesting that DNA methylation status of ctDNA relates with the clinical state, thereby representing potential early diagnostic or surveillance markers. In HPV-associated OPSCC, HPV ctDNA is also a potential marker for monitoring tumor recurrence, treatment response, and diagnosis [104]. Hence, combining these markers might be more useful to develop a new treatment strategy.

## 9. Targeted Therapy for DNA Methylation

Epigenetic-targeted therapy, especially targeting DNMTs, has the potential for tackling HPV-associated HNSCC. 5-azacytidine and 5-aza-2′-deoxycytidine are the most used, U.S. Food and Drug Administration-approved drugs (2004 and 2006, respectively) [105,106,107]. These drugs are cytidine analogs that are incorporated into DNA, leading to covalent adduct formation and working as DNMTs inhibitors [108]. They are used only for the treatment of some myelodysplastic syndrome and chronic myelomonocytic leukemia cases, and their efficacy for solid cancers is under consideration [109], with clinical trials still ongoing. One clinical trial is currently using 5-azacytidine for the treatment of HPV-associated and HPV-negative HNSCC (NCT02178072). HPV-associated HNSCC tends to have higher methylation levels compare with HPV-negative HNSCC; thus, these drugs might be good candidates for treating HPV-associated HNSCC.

## 10. Conclusions

HPV induces DNA methylation in a complex manner during carcinogenesis. In this review, we provided an overview of DNA methylation, the relationship between DNA methylation and HPV-associated HNSCC, and how these mechanisms are related to the carcinogenesis of HPV-associated HNSCC. Recent comprehensive large-cohort DNA methylation analyses at a genome-wide scale have revealed that there is an HPV-associated HNSCC subtype with increased DNA methylation. However, there is still room for elucidation of the mechanism of HPV and DNA methylation. A more detailed understanding of the molecular basis of this subtype might lead to the development of new therapeutic strategies, such as therapeutic de-escalation in this subtype.

## Figures and Tables

**Table 1:** Frequently hypermethylated genes in human papillomavirus-associated head and neck squamous cell carcinoma.

Function or Signaling	Gene	Location	Reference
Cell cycle	*CCNA1*	13q13.3	[27,55,56]
Apoptosis	*RASSF1*	3q21.31	[57]
Cellular adhesion	*CDH1* *CDH8* *CDH11 CDH13* *CDH15 CDH18* *CDH19 CDH23* *ITGA4*	16q22.1 16q21 16q21 16q23.3 16q24.3 5p14.3 18q22.1 10q22.1 2q31.3	[56,58,59,60,61,62]
Cellular migration	*TIMP3*	22q12.3	[60]
Differentiation	*CTNNA2* *RXRG* *GATA4*	2p12 1q23.3 8p23.1	[58,63]
GPCR signaling	*GHSR CASR* *NMUR1 PTGDR1PTGDR2 PTGIR*	3q26.2 3q21.1 2q37.1 14q22.1 11q12.2 19q13.32	[58,64,65]

**Table 2:** DNA methylation analysis of a small sample cohort on a genome-wide scale using human papillomavirus (HPV)-associated head and neck squamous cell carcinoma.

Total # of Samples	# of HPV (+) Samples	# of HPV (−) Samples	Type of Sample	Type of Analysis	Key Finding	Reference
32 HNSCC samples	18	14	FFPE and FF	Infinium 450 k	Polycomb repressive complex 2 target genes, including *CDH1*, were hypermethylated in the HPV (+) cases	[81]
13 OPSCC samples	4	9	FF	Infinium 450 k	Hypermethylation of *RXRG*, *GHSR*, *CTNNA2*, and *ITGA4* was significantly correlated with the HPV (+) status	[80]
35 HNSCC samples	35	0	FF	Infinium 450 k	*BARX2* and *IRX4* were hypermethylated and repressed in integration-negative tumors	[88]
15 OPSCC samples	5	10	FF	CpG-island tiling array	*ALDH1A2, OSR2, GATA4, GRIA4,* and *IRX4* were significantly hypermethylated in the HPV (+) HNSCC samples	[85]

FFPE: Formalin-fixed paraffin-embedded, FF: fresh frozen.

**Table 3:** Comprehensive large-cohort DNA methylation analysis at a genome-wide scale using human papillomavirus (HPV)-associated head and neck squamous cell carcinoma.

Total # of Samples	# of HPV (+) Samples	# of HPV (−) Samples	Type of Sample	Type of Analysis	Key Finding	Reference
279 HNSCC samples	36	243	FF	Infinium 450 k	No HPV (+) subtype	TCGA [23]
528 HNSCC samples	99	429	FF	Infinium 450 k	One HPV (+) subtype out of five groups	[90]
326 HNSCC samples	63	263	FF and FFPE	Infinium 450 k	One HPV (+) subtype out of three groups	[89]
101 OPSCC samples	101	0	FF	MBD-seq and Infinium 450 k	Three HPV (+) subtypes, including one high DNA methylation subtype with *CREBBP* mutation	[91]
170 OPSCC samples	107	63	FF	Infinium 450 k	Two HPV (+) subtypes, including one high DNA methylation subtype with a significantly good prognosis	[83]

FF: Fresh frozen, FFPE: formalin-fixed paraffin-embedded, MBD-seq: methyl-binding protein domain sequencing.

## Data Availability

Not applicable.
